# Can a Fraction of Flour and Sugar Be Replaced with Fruit By-Product Extracts in a Gluten-Free and Vegan Cookie Recipe?

**DOI:** 10.3390/molecules29051102

**Published:** 2024-02-29

**Authors:** Carlotta Breschi, Silvia D’Agostino, Francesco Meneguzzo, Federica Zabini, Jasmine Chini, Luca Lovatti, Luca Tagliavento, Lorenzo Guerrini, Maria Bellumori, Lorenzo Cecchi, Bruno Zanoni

**Affiliations:** 1 Department of Neurosciences, Psychology, Drug Research and Child Health (NEUROFARBA), University of Florence, 50121 Florence, Italy; carlotta.breschi@unifi.it (C.B.); maria.bellumori@unifi.it (M.B.); 2 Institute of Bioeconomy, National Research Council, 50019 Florence, Italy; francesco.meneguzzo@ibe.cnr.it (F.M.); federica.zabini@ibe.cnr.it (F.Z.); 3 Department of Agriculture, Food, Environment and Forestry Sciences and Technologies (DAGRI), University of Florence, 50121 Florence, Italy; silvia.dagostino@unifi.it (S.D.); bruno.zanoni@unifi.it (B.Z.); 4 R&D Department, Consorzio Melinda Sca, Via Trento 200, 38023 Cles, Italy; jasmine.chini@melinda.it (J.C.); luca.lovatti@melinda.it (L.L.); 5 HyRes S.r.l., Via Salvator Rosa 18, 82100 Benevento, Italy; luca.tagliavento@hyres.it; 6 Department of Land, Environment, Agriculture and Forestry (TESAF), University of Padova, 35122 Padua, Italy; lorenzo.guerrini@unipd.it

**Keywords:** vegan, food waste, by-products, fortification, polyphenols, hydrodynamic cavitation, antioxidants, pectin, cookies, gluten-free

## Abstract

Certain food by-products, including not-good-for-sale apples and pomegranate peels, are rich in bioactive molecules that can be collected and reused in food formulations. Their extracts, rich in pectin and antioxidant compounds, were obtained using hydrodynamic cavitation (HC), a green, efficient, and scalable extraction technique. The extracts were chemically and physically characterized and used in gluten-free and vegan cookie formulations to replace part of the flour and sugar to study whether they can mimic the role of these ingredients. The amount of flour + sugar removed and replaced with extracts was 5% and 10% of the total. Physical (dimensions, color, hardness, moisture content, water activity), chemical (total phenolic content, DPPH radical-scavenging activity), and sensory characteristics of cookie samples were studied. Cookies supplemented with the apple extract were endowed with similar or better characteristics compared to control cookies: high spread ratio, similar color, and similar sensory characteristics. In contrast, the pomegranate peel extract enriched the cookies in antioxidant molecules but significantly changed their physical and sensory characteristics: high hardness value, different color, and a bitter and astringent taste. HC emerged as a feasible technique to enable the biofortification of consumer products at a real scale with extracts from agri-food by-products.

## 1. Introduction

In recent decades, food loss and food waste have become a worldwide problem due to the increasing burden of material requiring disposal and the loss of valuable bioactive compounds. Many authors have well described the problems related to food loss/waste and highlighted the importance of innovative approaches and methods to exploit the waste resources [1,2,3]. Food loss/waste is rich in bioactive compounds such as fiber, vitamins, minerals, phenolic compounds, and other molecules with health-promoting and technological properties [4,5]. In the field of bioactive compounds from widely available food waste resources, three points are key: (i) which food wastes are richest in powerful compounds; (ii) which extraction techniques are the most promising and have the lowest ecological footprint; (iii) what are the applications (e.g., food industry, cosmetic industry) to boost their value. Food “enrichment” and/or “biofortification” with molecules extracted from food by-products are more and more embraced by consumers. In the “circular economy” idea, the use of molecules and extracts from food by-products is well accepted by consumers and markets, especially if these molecules have proven beneficial effects on health. Furthermore, such molecules can be used to improve the technological characteristics of food, especially for products that are more difficult to prepare such as gluten-free or special diet products (e.g., vegetarian or vegan). In order to be consistent with economic and environmental sustainability, it is desirable that the food losses/wastes used for the extraction of bioactive molecules represent an actual problem in the reference production sector. This happens for largely consumed produce like fruits, as the transformation processes of fruit-based foods (juices, jams, purées, etc.) produce a large amount of food loss/waste. 

In this context, apples are some of the most consumed fruits worldwide. Therefore, waste streams from the apple production chain (e.g., apples unsuitable for the market, residues from juice squeezing known as apple pomace) are well studied due to the high availability of pectin and several antioxidant compounds such as phenolic compounds [2,6,7,8]. The considerable number of by-products resulting from the industrial processing of apples, starting from apples unsuitable for sale, has received increasing interest due to the availability of vitamins, phenolic compounds (e.g., phenolic acids), and flavonoids, which are responsible for the antioxidant activity of the fruit [9,10]. The two main topics that several studies focused their attention on are the best applications of both by-products and their extracts, as well as the extraction techniques and related methods. On the first side, valuable applications were identified such as biofortification and functionalization of foods including bakery products, fish- and meat-derived products, and functional packaging bio-materials. On the second side, few technologies were evaluated based on functional metrics such as extraction yield and the recovery and preservation of bioactive compounds available in the raw material. It is possible to distinguish conventional and non-conventional or innovative extraction technologies [11]. The best-known conventional extraction methods are maceration, magnetic stirring, Soxhlet extraction, and hydro-distillation. Although conventional solid–liquid, heat-assisted extraction methods guarantee good extractive yields, they all suffer from specific drawbacks due to the use of solvents, insufficient control, and repeatability, decreasing convenience and feasibility with upscaling to the industrial level. Furthermore, the extraction parameters sometimes favor the yield of one bioactive compound to the detriment of another: in apples, polysaccharide extraction is favored when long times, high temperatures, and hydro-acidic environments are applied, while the phenolic fraction requires less time and generally low heat to avoid thermal degradation [12,13,14]. Therefore, new and greener technologies were investigated, for example, ultrasound-assisted extraction, microwave-assisted extraction, pressurized liquid extraction, and supercritical fluid extraction. However, although quite successful at the laboratory scale, none of them has so far been demonstrated at scales representative of industrial production rates.

Pomegranate peel and seeds represent another widespread side stream, resulting from juice squeezing of the pomegranate fruit accounting for an average of 50% of the weight of the whole fruit [15]. Pomegranate peel and seeds are rich in primary and secondary metabolites, many of which are endowed with powerful healthy activity, such as pectic polysaccharides, polyphenols such as ellagitannins, punicalagin, catechin, gallocatechin, anthocyanins and procyanidins, and unsaturated fatty acids [16,17,18,19,20]. Extraction is a key step to optimally recover these bioactive compounds; polysaccharides and phenols of pomegranate peel and seeds are conventionally extracted in hot water or other polar solvents, since tannins are more stable at higher temperatures, while fatty acids need nonpolar solvents such as hexane to be dissolved. Phenols can also be extracted from the peel through innovative techniques, mostly ultrasound- and microwave-assisted extraction. Moreover, to extract less polar compounds such as ellagic acid in the peel and fatty acids from the seeds, supercritical fluid-assisted extraction is also effective [21,22,23,24]. However, as in the case of apple by-products, none of these technologies could meet the requirements of large-scale production.

In recent years, scholars turned to the assessment of further efficient, green, and scalable extraction techniques, among which hydrodynamic cavitation (HC) stood out in terms of extraction rate, efficiency, and scalability, as shown, for example, with the application to the extraction of whole pomegranate fruit [25]. HC is a green extraction electricity-powered technique requiring water as the only solvent, operating at atmospheric pressure and generally at low temperatures, and affording fast extraction of both hydrophilic and lipophilic compounds from raw materials [26,27,28,29]. HC-driven, real-scale extraction of biomolecules from food by-products has already been the subject of numerous studies, and the results highlighted very good performances in the extraction of pectin, cellulose, starch, and antioxidant compounds such as phenols and tannins from both food products and by-products [28,30,31] and forestry by-products [32].

The use of extracts obtained using HC in consumer goods has been tested, for example, in gluten-free biscuits supplemented with a pectin-based compound extracted from waste lemon peel [33] and whole-wheat bread supplemented with an extract from silver fir needles [34]. However, the technological properties of these extracts have never been investigated. 

The aim of this study was to assess if part of the flour and sugar in the recipe of gluten-free and vegan cookies could be replaced with extracts from food by-products such as whole apples unsuitable for sale and pomegranate peel. To this aim, whole apple and pomegranate peel extracts were obtained using HC, and their technological role in the recipe was investigated, particularly focusing on whether the molecules contained in the extracts can improve or maintain the characteristics of widely consumed products for special diets. The choice of whole apples unsuitable for the market, instead of apple pomace, was motivated by the far easier preservation of the former, while apple pomace is rapidly degraded by the action of the abundant polyphenol oxidize enzyme [11]. 

## 2. Results and Discussion

### 2.1. Chemical Characterization of Extracts

The whole apple extract (AE) and pomegranate peel extract (PPE) samples were characterized for the nutritional label with data reported in Table 1. The AE was characterized by a calorie content higher than PPE, and the calorie content of both samples was due to the carbohydrate content. However, in the AE sample, all the carbohydrates (58.1 g/100 g) are sugars (58.1 g/100 g). Instead, in the PPE sample, the sugar content is about half (8.7 g/100 g) of the total carbohydrate content (16.0 g/100 g). Moreover, the AE sample contains a dietary fiber content higher than PPE and a total protein content lower than PPE.

The total phenolic content (TPC) and DPPH (2,2-diphenyl-1-picrylhydrazyl) radical-scavenging activity, reported as IC_50_ (half maximal inhibitory concentration in µg/mL), were also determined in AE and PPE. The PPE sample was characterized by a TPC of 161.3 mgGAE/g and an IC_50_ of 17.3 µg/mL. The AE sample was characterized by a lower TPC (10.2 mgGAE/g), and a higher IC_50_ (949.0 µg/mL). Besides the well-known very high content of antioxidant compounds in pomegranate peel, based on Table 1, an important contribution to these results derives from the far higher contents of carbohydrates and fiber in the AE sample (about 68% of total mass, against about 16% for PPE), which do not contribute to TPC and DPPH-scavenging activity.

### 2.2. Physical Properties of Cookies Immediately after Baking

The physical properties of cookies immediately after baking were determined to observe whether the whole apple extract and pomegranate peel extract obtained from the hydrodynamic cavitation technique could mimic the role of a sugar and flour part.

After baking, all cookie samples presented the following values: a final weight between 10 and 11 g, a final moisture content between 2.8 and 4.8%, and a final water activity between 0.16 and 0.29, with no statistically significant differences (*p*-value > 0.05) (Table S1).

Table 2 shows the diameter, thickness, and spread ratio of cookie samples: CON indicates the control cookie recipe; A05, P05, A10, and P10 indicate the cookie recipe in which the amount of flour and sugar was replaced with AE or PPE to a level of 5% (A05 and P05) and 10% (A10 and P10) in the cookie recipe; W05 and W10 indicate the cookie recipe in which 5% (W05) and 10% (W10) of flour and sugar were removed but not replaced. The differences in these parameters are related to ingredient properties and interactions [35]. 

The differences in diameters across different samples are statistically significant (*p*-value = 0.002). The diameter of cookies is affected by gravitational flow, which depends mostly on sugar dissolving in water during baking: the more dissolved sugar, the higher the gravitational flow [36,37,38]. Although AE is less soluble than PPE (see Section 3), the A10 sample was found to be larger (6.1 ± 0.1 cm) than other samples, followed by A05 (5.9 ± 0.2 cm). P05 was the tighter sample (5.5 ± 0.1 cm), although it was non-significantly different from P10 and W05. The greater increase in diameter in samples containing AE may be due to two phenomena: (i) AE and PPE were manually dissolved in water before being added to the recipe, and this makes AE behave as if it were more soluble than it is, increasing the viscosity of dough [37,39,40,41]; (ii) AE is an extract rich in sugars. Because the substitution is composed of one-third sucrose and two-thirds flour, the total sugar content removed for the substitution was 1.09/3, considering both the sugar of the recipe and the sugar content of the gluten-free flour (3 g/100 g). The apple extract is characterized by a sugar content of 58.1 g/100 g of extract (Table 1). When the apple extract is added to the A05 and A10 samples, the total content of sugar is higher (1.74/3) than the total content of sugar removed (1.09/3) from the CON sample. The increase in sugar content in the cookies with AE may have led to greater sugar syrup formation during baking, with a consequent increase in gravitational flow, resulting in larger cookies [35,42,43]. 

In the literature, the thickness of cookies is related to gluten development, the expansion of dough by leavening, and the amount and type of sugar in the recipe [35,36,38], which is not applicable to gluten-free recipes. However, the gluten-free flour used for the recipe contains hydroxypropyl methylcellulose as a thickener, which gives structure to gluten-free baked goods [44]. In this study, the thickness of cookies decreased statistically significantly (*p*-value = 0.006) with the substitution of flour and sugar with extracts, and it was due to the extract concentration. Samples A10 and P10 had the lowest height (0.91 ± 0.03 cm and 0.93 ± 0.04 cm, respectively), followed by A05 and P05 (both 0.99 ± 0.08 cm), W05 and W10 (1.05 ± 0.03 cm and 1.05 ± 0.02 cm, respectively), and the CON sample, which had the greatest height (1.11 ± 0.03 cm) (Table 2). As described for the diameter, AE and PPE were manually dissolved in water before being added to the recipe. This operation made the water present in the recipe less available for the formation of the gluten-free structure, resulting in thinner cookies than CON [35,36].

The ratio between diameter and thickness is the spread ratio, and it depends on which phenomena previously described prevail. The spread ratio is one of the most important quality parameters of biscuits and cookies because it is correlated with texture, overall mouthfeel of the cookies, and consumers’ acceptability and preferences [35,38,45,46,47,48,49]. The CON sample had the lowest spread ratio (5.2 ± 0.2), related to the higher thickness and medium diameter. Although the decrease in sugar and flour content in W05 (5.3 ± 0.1) and W10 (5.6 ± 0.2) led to an increase in spread ratio compared to CON, the differences were not significant. The spread ratio of cookies increased statistically significantly (*p*-value < 0.001) with the substitution of flour and sugar with extracts, and it was dependent on the concentration of the extracts. A10 had the highest spread ratio value (6.7 ± 0.3), related to the larger diameter and the smaller thickness, followed by P10 (6.2 ± 0.3), A05 (6.0 ± 0.3), and P05 (5.6 ± 0.4) (Table 2). 

**Figure 1:**
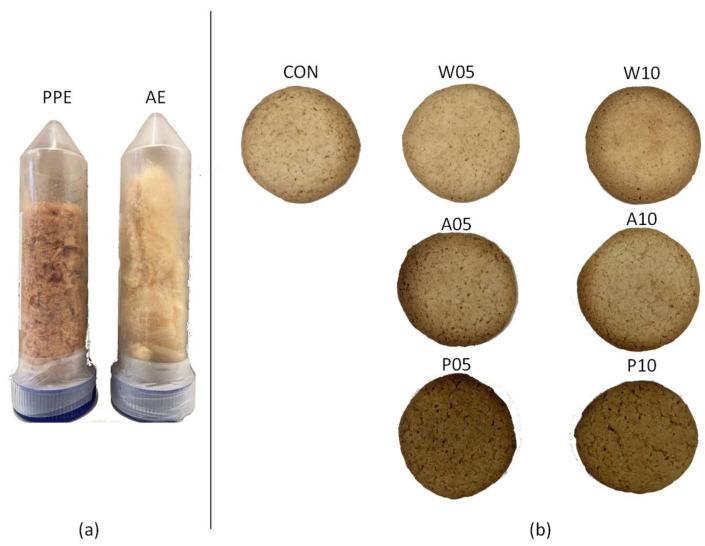
(**a**) Pomegranate peel extract and apple extract; (**b**) Cookie samples.

In Table 2, the lightness (L*), redness index (a*), and yellowness index (b*) of the cookies’ upper surface were reported. The differences in sugar content and type, associated with the Maillard reaction [42,43], did not significantly affect the color of the cookies. However, significant differences (*p*-value < 0.001) in the lightness and redness indexes of the P05 and P10 samples are shown in Table 2 and Figure 1. P05 and P10 resulted in redder (7.8 ± 0.2 and 7.7 ± 1.0, respectively) and less bright (57.8 ± 0.5 and 56.7 ± 2.2, respectively) cookies than all the other samples. The addition of the red-colored PPE influences the cookies’ chromatics more than the addition of the yellow-colored AE (Figure 1) [43].

The statistically significant (*p*-value = 0.033) differences in cookie hardness are reported in Table 2. P05 (41.0 ± 10.7 N) and P10 (35.6 ± 7.0 N) were the hardest cookies, followed by A10 (33.5 ± 7.7 N), A05 (29.4 ± 1.9 N), W10 (25.5 ± 6.2 N), W05 (23.0 ± 4.2 N), and CON (23.4 ± 4.0 N). As in the case of the cookies’ dimensions, hardness is influenced by the ingredients’ properties and interactions. The formation of a gluten network—or, in this case, a gluten-free flour network—is inversely related to hardness: the higher the structural network, the lower the cookie’s hardness. As discussed above, the gluten-free structure formation is influenced by sugar presence because sugars preferentially attract water, making it less available for network formation, with a consequent increase in hardness [35,41,43]. This behavior explains the high hardness value of A10 and A05, which are characterized by a higher sugar content than other samples (Table 2). Moreover, sugars of AE recrystallize quickly due to the natural low solubility of AE, leading to a redistribution of water to other ingredients, with a consequent increase in hardness [36]. Although the whole apple extract is not comparable to apple pomace, the result obtained is in line with the study by Kruczek et al. [50], in which the hardness increases with the content of apple pomace due to both sugar and dietary fiber content. Indeed, the fiber content in AE (9.6 g/100 g) is higher than the fiber content of the gluten-free flour employed (1.6 g/100 g); thus, the dietary fiber of AE can further influence the increase in the hardness of cookies [50,51]. Although PPE is poor in sugars and fibers, the hardness is higher in P05 and P10 than in other samples. Nuzzo et al. [52] showed that the use of pomegranate peel powder in cookie preparations increased the viscosity of the dough and facilitated the lamination. Nevertheless, an increase in dough viscosity is positively associated with the hardness of cookies [37,40,41].

In the literature, the spread ratio value is often inversely related to hardness: the higher the spread ratio, the lower the hardness [35,36,37,41,43,53,54]. In this work, the relationship is reversed due to the combined effect of the components of the extract (sugars, fibers, proteins) and the dissolution of the extract in water before mixing, which allowed the extract to behave as if it were more soluble during cooking.

### 2.3. Chemical Properties of Cookies Immediately after Baking

The chemical properties immediately after baking were determined to observe whether the whole apple extract and pomegranate peel extract obtained from the hydrodynamic cavitation technique could “enrich” the cookies with bioactive molecules, which lead to beneficial effects on health.

The TPC and DPPH radical-scavenging activities in cookies were determined the day after baking and the results are reported in Table 3. The substitution of flour + sugar with extracts led to an increase in TPC in baked cookies. The highest and statistically significant (*p*-value < 0.001) levels of TPC were found in P10 (9.77 ± 0.95 mgGAE/g), followed by P05 (6.15 ± 0.37 mgGAE/g). Samples A05 and A10 had a TPC higher than CON, W05, and W10, but it was non-statistically significant (*p*-value > 0.05) (Table 3). As confirmed by previous studies [52], the high value of TPC in P05 and P10 is due to the high value of TPC in PPE (161.3 mgGAE/g).

The DPPH radical-scavenging activity, reported as IC_50_ (µg/mL), showed statistically significant differences (Table 3). The substitution of flour + sugar with an extract rich in phenols such as PPE led to a significant (*p*-value < 0.001) decrease in the IC_50_ value in P05 (3.1 ± 1.9 µg/mL) and P10 (1.2 ± 0.3 µg/mL), but the difference between the levels in P05 and P10 were not statistically significant. The samples A05 (28.9 ± 3.9 µg/mL) and A10 (20.7 ± 0.9) were significantly different from each other, suggesting a dose-dependent effect, but were not significantly different from CON (25.6 ± 4.3 µg/mL), thus confirming the lower antioxidant activity of AE. The removal of 5% and 10% of flour + sugar led to a significant increase in the IC_50_ value because of the composition of the gluten-free flour used for the recipe. 

### 2.4. Sensory Evaluation of Cookies Immediately after Baking

The sensory evaluation of cookies immediately after baking was carried out to observe the effect of AE and PPE on the taste, aroma, flavor, and mouthfeel of cookies.

**Figure 2:**
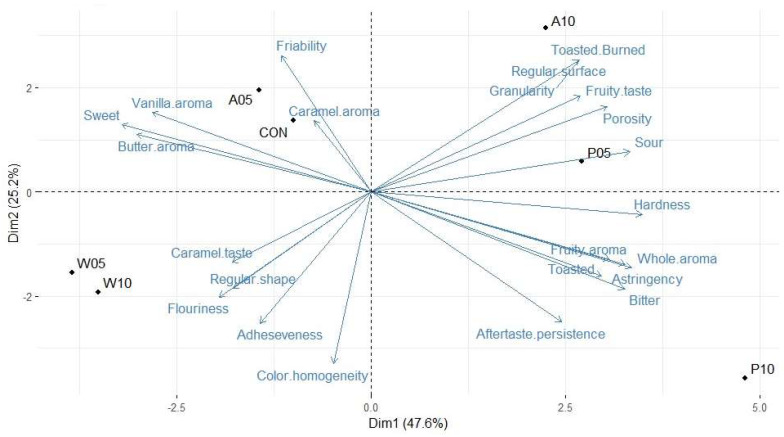
Principal Component Analysis (PCA). The descriptors are reported in blue and samples are reported in black.

The results obtained by the Quantitative Descriptive Analysis (QDA) method were processed according to a Principal Component Analysis (PCA), which allowed us to better compare the samples with each other and understand which characteristics best describe the cookies (Figure 2). The first two principal components accounted for 72.8% of the total variance (47.6% and 25.2%, respectively). 

The first component (Dim1) was positively correlated with hardness, sourness, astringency, bitter, fruity aroma, whole aroma, burned taste (reported as “Toasted.Burned”), and burned aroma, while sweet, vanilla, and butter aromas and caramel taste contributed to the negative side of Dim1. Friability and color homogeneity, respectively, showed a positive and negative correlation on the second component (Dim2). Samples W05 and W10 showed similar characteristics of caramel taste, flouriness, adhesive sensation in the mouth, sweet taste, butter aroma, and regular shape. Samples with PPE were described by the same attributes but with different intensities. P10 and P05 were described by the panel as hard, according to the textural hardness value, with higher intensities of whole aroma, fruity aroma, and taste. The high value of bitterness and astringency in P05 and P10 is due to the high content of phenolic compounds, particularly ellagitannins, in PPE. Moreover, P10 had the most persistent aftertaste value. 

Sample A10 was characterized by high friability, regular surface, and toasted aroma due to the high content of sugars: they re-crystallized during cooling and conducted the Maillard reaction. Sample A05 was found to be different from A10 but very similar to CON; both samples were described by the panel as sweet, with caramel, butter, and vanilla aromas and with high friability in the mouth; all these descriptors are well accepted and expected in a cookie by consumers. 

### 2.5. Physical and Chemical Properties after One Month of Conservation

During 30 days of conservation at room temperature conditions, the cookies showed no differences in moisture content, an increase (*p*-value > 0.05) in water activity value (Table S1) due to sugar crystallization, and a consequent releasing and redistribution of water inside the cookies [41,55]. Although the hardness of cookies showed no statistically significant differences (*p*-value > 0.05) over time, it was observed that cookies without extracts (CON, W05, and W10) had an increase in hardness, while cookies with AE and PPE (A05, A10, P05, and P10) had a decrease in hardness (Table S1). This different behavior may be due to the different sugar and fiber contents. 

Regarding the TPC and the DPPH radical-scavenging activity, no statistically significant difference (*p*-value > 0.05) during storage time was found (Table S2). The cookies supplemented with PPE (P05 and P10) kept high TCP and low IC_50_ levels during the conservation in glass jars, showing good antioxidant stability of the phenolic pomegranate extracts.

## 3. Materials and Methods

### 3.1. Samples

Whole apple fruits of the Renetta variety used in this study were supplied by Consorzio Melinda S.c.a. (Cles, Trento, Italy) in November 2022 and processed the day after their arrival. They were fruits that could not be marketed because their appearance did not comply with the sales criteria (e.g., damaged, non-uniform color).

Pomegranate fruits of the Wonderful One variety were purchased at a local market in the Apulia region, Salento area (southeastern Italy), in early October 2022, preserved in the dark at 4 °C, and processed a few days later. Whole fruits were cut in half and squeezed manually using a commercial home tool (Pomegranate/citrus fruit squeezer Model AAA0000982081, Ilsa, Collegno, Italy), and the juice was discarded. All the other parts were retained for extraction: crown, peel (constituted by exocarp and mesocarp), endocarp, and seeds, which are typical by-products of pomegranate processing.

Both whole apple fruits and pomegranate by-products were ground to coarse particles (5–20 mm) through a fruit mill (Model HP3, Polsinelli, Isola del Liri, Italy) and immediately processed.

#### 3.1.1. HC-Based Extraction of Whole Apple and Pomegranate Peel

The production of whole apple (AE) and pomegranate peel (PPE) aqueous extracts was performed by a semi-industrial-scale hydrodynamic cavitation (HC) pilot device, with a maximum payload of 200 L. The HC-based device was described in a previous study [30].

The relative humidity of the used biological materials was assessed by drying an amount of 0.5 kg of biomass overnight at 75 °C using a commercial device (Electric Food Dryer, model LT 85, Kwasyo, Zhongshan, China). A small amount of lemon juice was added to the water to counteract the oxidation resulting from fruit cutting and subsequent contact with oxygen. No active heat dissipation method was applied during the extraction process.

The basic features of the two extraction processes are shown in Table 4.

The aqueous extracts were filtered (rough-cut filter~15 µm), centrifuged at 4500 rpm (2722× *g*) for 30 min (NEYA 8xs, Remi Elektrotechnik Ltd., Palghar, India), frozen at −18 °C for 24 h, and freeze-dried for 48 h (Modulyo, Edwards, Milan, Italy). The powder of AE and PPE was collected in a 50 mL plastic tube and kept at −18 °C until use. 

#### 3.1.2. Physical and Chemical Characterization of the Extracts

The AE and PPE were characterized for their nutritional properties (nutrition label) by an external lab (Laboratorio Empolese di Analisi, Empoli (Firenze), Italy).

The water solubility index (WSindex) of the extracts was determined with the method described by Lisiecka et al. [56], with some modifications. In a 50 mL centrifuge tube, AE and PPE were suspended in water (at room temperature) at different percentage concentrations (30, 60, and 90%), stirred for 30 min, and centrifuged at 4500 rpm (2722× *g*) for 10 min (NEYA 8xs, Remi Elektrotechnik Ltd., Palghar, India). The supernatant was transferred into an aluminum dish and evaporated at 100 °C for 24 h in a vacuum oven (OVL570 010J, Gallenkamp Labs, A Division of Synoptics Ltd., Cambridge, UK). The remaining extract was weighed, and the WSindex was determined as the ratio of solids by weight in the supernatant after drying to the dry weight of the sample. The WSindex allowed determining the maximum concentration of extracts that should be used in the cookie recipe. With a concentration of 30% of AE and PPE in water, the WSindex was 81.7% and 81.1%, respectively. Increasing the extract concentration to 60% and 90% revealed differences between extracts: in comparison to the extract concentration of 30%, AE showed a WSindex decrease of 36.6% (WSindex value of 51.8%) and 42.6% (WSindex value of 46.9%) for the concentrations of 60% and 90%, respectively. In contrast, PPE showed a WSindex decrease of 0.6% (WSindex value of 80.6%) and 9.1% (WSindex value of 73.7%) for the concentrations of 60% and 90%, respectively. Based on the decrease in the WSindex of AE and the observed difficulty in completely dissolving AE at 60%, a maximum concentration of extracts of 60% of the water content was used in the cookie recipe.

The total phenolic content (TPC) in extracts was determined by means of the Folin–Ciocalteu assay [57]. An aliquot of 200 µL of diluted extract in water (0.001 g/mL) was added to 1.5 mL of freshly prepared Folin–Ciocalteu reagent (10-fold diluted). After 5 min of equilibration, 1.5 mL of sodium carbonate solution (60 g/L) was added to the mixture and incubated for 90 min at 25 °C. Absorbance was read at 725 nm (Lambda 35 UV/Vis Spectrometer, Perkin Elmer, Waltham, MA, USA). A gallic acid (GA) calibration curve was used to express the total phenolic content as mg GA equivalents/g of sample.

DPPH radical-scavenging activity was detected using the procedure described by Brand-Williams et al. [58], with some modifications. About 1 mL of diluted extract in water was reacted with 1 mL of 1 × 10^−4^ mol/L of DPPH solution. The absorbance was read at 517 nm after 0 and 20 min using a methanol blank (Lambda 35 UV/Vis Spectrometer, Perkin Elmer, Waltham, MA, USA). The DPPH radical-scavenging activity is reported as IC_50_ as described by Bonina et al. [59]. The percentage of DPPH remaining at a steady state was plotted against the antioxidant concentration to obtain the concentration of antioxidants necessary to decrease the initial concentration by 50% (IC_50_ µg/mL). 

### 3.2. Vegan and Gluten-Free Cookie Preparation

The vegan and gluten-free cookie samples were created using the AACC method 10–54.01 [60] with modifications. The control (CON) formula was made up as follows: 120 g all-purpose flour (Mix it! Universal, Schär, Bolzano, Italy), 60 g white sugar (Classico, Eridania, Italy), 2 g baking powder (Lievito Pane degli Angeli, Paneangeli, Italy), 48 g margarine (Vallé Pasticceria, Vallé, Italy), 30 g water at room temperature. Considering both the extracts’ WSindex and the water content used in the recipe (30 g), the maximum content of extracts allowed was 18 g, which corresponds to 10% of the amount of flour and sugar (120 g + 60 g). The amount of flour and sugar was replaced with AE or PPE to a level of 5% (A05 and P05) and 10% (A10 and P10) in the cookie recipe. The extracts were taken out of the fridge (−18°C) at the time of use. More cookies were produced in which 5% (W05) and 10% of flour and sugar were removed but not replaced (Table 5) to evaluate whether the differences observed were due to the presence of the extracts or to the absence of the amount of flour and sugar removed from the recipe.

All dough ingredients were mixed in a mixer-type KHC29 (Kenwood Ltd., Woking, UK) equipped with a K beater. First, all-purpose flour, sugar, and baking powder were mixed with a whisk, then margarine was taken out of the fridge and added and mixed in the mixer using the sanding method (approx. 1 min). Finally, water was added, and the dough was mixed for 2 min. In A05, A10, P05, and P10, the extracts were dissolved in water and then added to the dough. Then, the dough was rolled out with an adjustable rolling pin to obtain a 6 mm thickness and cut out using a mold with a diameter of 5 cm. Cookies were baked in an electric oven (Cuocitutto, DPE Elettrodomestici, Brescia, Italy) at 170 °C for 16 min. After cooling (approx. 3 h at room temperature), the samples were put in glass jars and stored for 1 month at room temperature to simulate home storage. Each sample was made in triplicate.

Texture, water activity, moisture content, total phenolic content, and DPPH radical-scavenging activity were analyzed the day after baking (t0) and after 1 month of storage (t1). Additionally, weight, thickness, diameter, spread ratio, color, and sensory properties were determined the day after baking.

### 3.3. Physical Properties

#### 3.3.1. Weight, Dimension and Color Analysis

The cookies were weighed in quintuplets and the average was reported. The diameter and thickness of samples were measured as described by Hamdani et al. [41] using a steel precision ruler, and the spread ratio was determined as the ratio between diameter and thickness [60]. 

The instrumental color measurements were performed on the cookies’ upper surface, in a double-opposite position, with a Chroma Meter CR-400 (Konica Minolta, Chiyoda, Japan). The color was collected as lightness (L*), redness index (a*), and yellowness index (b*) according to the CIELAB system (CIE, 1976). Five cookies were evaluated for each formulation.

#### 3.3.2. Water Activity, Moisture Content, and Texture (Textural Hardness)

Water activity (Aw) at 25 °C was measured using a Rotronic Hygroskop DT hygrometer (Process Sensing Technologies PST Srl, Milan, Italy), previously calibrated with a known salt solution (Rotronic, Process Sensing Technologies PST Srl, Milan, Italy). Approx. 2 g of cookie sample, finely crumbled by using a pestle and a mortar, was used for the measurement. Moisture content (%) was measured using the method described by AACC 44-15.02 [61].

Texture measurements were performed using a Zwick Roell^®^ 109 texturometer (Zwick GmbH & Co. KG, Ulm, Germany) equipped with a 1 kN load cell and an adapted Warner–Bratzler shear blade (width of 7 cm), setting the crosshead speed at 30 mm min^−1^. The blade was then pressed through an intact cookie sample. Data were collected and analyzed by the Test-Xpert2 by Zwick Roell^®^ software version 3.0. Hardness, reported in Newtons (N), is given by the peak force (Fmax) required to break the cookies [41]. For each formulation, seven cookies were evaluated, two extreme results were rejected, and the remaining five were used to calculate the arithmetical mean (Kruczek, 2023) [50].

### 3.4. Total Phenolic Content and DPPH Radical Scavenging Activity

Total phenolic content (TPC) was extracted and determined using the method described by Gao et al. [62]. Cookies were finely crumbled by means of a pestle and a mortar, and 2 g of sample was extracted with 40 mL of acidified methanol (HCl/water/methanol, 1:10:80 *v*/*v*/*v*) at room temperature for 2 h under magnetic stirring and centrifuged (NEYA 8xs, Remi Elektrotechnik Ltd., Palghar, India) for 20 min at 4500 rpm (2722× *g*). An aliquot of 200 µL supernatant was added to 1.5 mL of freshly prepared Folin–Ciocalteu reagent (10-fold diluted). After 5 min of equilibration, 1.5 mL of sodium carbonate solution (60 g/L) was added to the mixture and incubated for 90 min at 25 °C. Absorbance was read at 725 nm (Lambda 35 UV/Vis Spectrometer, Perkin Elmer, Waltham, MA, USA). A gallic acid (GA) calibration curve was used to express the total phenolic content as mg GA equivalents/g of sample.

DPPH radical-scavenging activity was determined using the extraction described by Hamdani et al. [41]. The cookies were crumbled, and 1 g of sample was added to 10 mL of methanol, sonicated at room temperature for 2 h, and centrifuged for 15 min at 4500 rpm (2722× *g*). About 1 mL of supernatant was reacted with 1 mL of 1 × 10^−4^ mol/L of DPPH solution, and the DPPH procedure described by Brand-Williams et al. [58], with some modifications, was used as reported above.

### 3.5. Sensory Analyses

A trained sensory panel evaluated the intensity of the sensory attributes of the seven cookie samples (CON, W05, W10, A05, A10, P05, P10) using the Quantitative Descriptive Analysis (QDA) method [63], with some modifications. During three training sessions using both commercial gluten-free and/or vegan cookies and the control cookie recipe, eight panelists—three males and five females aged between 25 and 45 years old—who had experience in quality evaluation of bakery products generated 23 descriptors for the categories of appearance, aroma, taste, flavor, texture/mouthfeel, and aftertaste (Table S3). In addition, panelists were asked to align concepts and select words to anchor each nine-point scale. The samples were identified with three-digit code numbers and presented in a random order to the panelists [43].

### 3.6. Data Processing

All the data are reported as the average of triplicates with standard deviation. The data relating to the cookies’ properties were statistically processed according to a one-way analysis of variance (ANOVA) and a multi-way ANOVA: the former was employed to compare the cookies immediately after baking and the latter to compare them during storage time. Jamovi 2.3.21 (JASP Project) was used for the data processing. The results of sensory evaluation were processed according to a Principal Component Analysis (PCA) using R software version 4.3.1 (R Foundation for Statistical Computing, Vienna, Austria).

## 4. Conclusions

The molecules contained in extracts of food by-products obtained using the green, efficient, and scalable HC technique can be used to “enrich” and/or “fortify” consumer goods for special diets (gluten-free, vegan).

This study focused on the use of not-good-for-sale whole apple extract and pomegranate peel extract in gluten-free and vegan cookies. The use of whole apple extract as a replacer of part of the flour + sugar allowed us to obtain cookies with better or similar characteristics compared to the control cookies. The A10 and A05 samples turned out to have a high spread ratio value, usually related to increased consumer acceptability. Moreover, the results showed that the cookies containing whole apple extract and the control cookies had similar color, in addition to similar good sensory characteristics—especially in the case of the A05 and CON samples. Therefore, the molecules in AE (including sugars and fiber) can mimic the role of the removed part of flour + sugar, resulting in good natural ingredients. On the other hand, the pomegranate peel extract provided the cookies with natural and stable antioxidant molecules. However, the use of PPE in the recipes significantly altered the physical and sensory characteristics of cookies: P05 and P10 appeared more red than CON, with high hardness values, and were perceived by panelists as bitter, astringent, sour, and hard. It is suggested that the use of pomegranate peel extracts be rethought, either by reducing the dose or redirecting it towards products of a different nature and structure, such as juices. 

Despite the positive results obtained with the supplementation of whole apple and pomegranate peel extracts in gluten-free and vegan cookie formulations, further studies on other by-products and formulations/different food products are necessary to identify the best-performing use of this technology from a “circular economy” perspective. Once the best by-products and the best formulation/food product have been identified, it would be appropriate to carry out an economic evaluation and a lifecycle assessment.

## Figures and Tables

**Table 1:** Nutrition label of whole apple extract (AE) and pomegranate peel extract (PPE).

	AE	PPE
Calories (kcal/100 g)	246	79
Calories (kJ/100 g)	1043	335
Total fats (g/100 g)	0.9	<0.1
Saturated fats (g/100 g)	0.4	<0.1
Total carbohydrates (g/100 g)	58.1	16.0
Total sugars (g/100 g)	58.1	8.7
Dietary Fiber (g/100 g)	9.6	0.2
Total proteins (g/100 g)	1.3	3.7

**Table 2:** Dimensions (diameter and thickness), spread ratio, CIELab values (L* = lightness index, a* = redness index, b* = yellowness index), and hardness of all samples.

	Diameter (cm)	Thickness (cm)	Spread Ratio	L*	a*	b*	Hardness (N)
CON	5.8 ± 0.2 ^b^	1.11 ± 0.03 ^a^	5.2 ± 0. 2 ^d^	73.8 ± 3.7 ^a^	4.1 ± 2.2 ^b^	27.9 ± 3.6	23.4 ± 4.0 ^b^
W05	5.6 ± 0.1 ^bc^	1.05 ± 0.03 ^ab^	5.3 ± 0.1 ^d^	71.8 ± 3.2 ^a^	4.2 ± 1.1 ^b^	25.1 ± 2.4	23.0 ± 4.2 ^b^
W10	5.8 ± 0.1 ^b^	1.05 ± 0.02 ^ab^	5.6 ± 0.2 ^cd^	74.6 ± 1.9 ^a^	3.1 ± 1.4 ^b^	25.7 ± 1.5	25.5 ± 6.2 ^ab^
A05	5.9 ± 0.2 ^ab^	0.99 ± 0.08 ^bc^	6.0 ± 0.3 ^bc^	73.9 ± 0.7 ^a^	2.8 ± 0.1 ^b^	26.9 ± 0.6	29.4 ± 1.9 ^a^
A10	6.1 ± 0.1 ^a^	0.91 ± 0.03 ^c^	6.7 ± 0.3 ^a^	69.5 ± 2.9 ^a^	3.9 ± 0.6 ^b^	25.7 ± 3.6	33.5 ± 5.7 ^a^
P05	5.5 ± 0.1 ^c^	0.99 ± 0.08 ^bc^	5.6 ± 0.4 ^cd^	57.8 ± 0.5 ^b^	7.8 ± 0.2 ^a^	32.1 ± 7.7	41.0 ± 10.7 ^a^
P10	5.7 ± 0.1 ^bc^	0.93 ± 0.04 ^c^	6.2 ± 0.3 ^b^	56.7 ± 2.2 ^b^	7.7 ± 1.0 ^a^	32.3 ± 7.7	35.6 ± 7.0 ^a^
*p*-value	**	**	***	***	***	ns	*

Results are expressed as means (n = 3) ± standard deviation. *, **, and *** indicate significant differences by one-way ANOVA at *p* < 0.05, *p* < 0.01, and *p* < 0.001, respectively, for the samples; different letters (a, b, c, d) indicate a statistically significant difference of the main effects with the Tukey HSD post hoc test (*p*-value < 0.05). ns = not significant.

**Table 3:** Total phenolic content (TPC) and DPPH as IC_50_ of all samples.

	TPC (mgGAE/g)	DPPH (IC_50_ µg/mL)
CON	0.75 ± 0.35 c	25.6 ± 4.3 bc
W05	0.61 ± 0.02 c	50.4 ± 6.5 a
W10	0.59 ± 0.10 c	46.9 ± 2.6 a
A05	0.92 ± 0.18 c	28.9 ± 3.9 b
A10	1.30 ± 0.12 c	20.7 ± 0.9 c
P05	6.15 ± 0.37 b	3.1 ± 1.9 d
P10	9.77 ± 0.95 a	1.2 ± 0.3 d
*p*-value	***	***

Results are expressed as means (n = 3) ± standard deviation. *** indicates significant differences by one-way ANOVA at *p* < 0.001 for the samples; different letters (a, b, c, d) indicate a statistically significant difference of the main effects with the Tukey HSD post hoc test (*p* < 0.05).

**Table 4:** Basic features of the extraction processes of AE and PPE. Specific energy is the electric energy consumed per unit dry mass of the raw material.

Extract Name	Water (L)	Lemon Juice (L)	Fresh Raw Material (kg)	Relative Humidity (%)	Time (min)	Temperature Range (°C)	Specific Energy (kWh/kg DW)
AE	44	5	96.9	84.3	117	24.0–78.0	0.852
PPE	108	2	47.3	75.0	20	31.0–39.0	0.216

**Table 5:** Cookie recipes. CON: control sample. W: cookies in which 5% (W05) and 10% (W10) of flour and sugar were removed but not replaced. A: cookies in which 5% (A05) and 10% (A10) of flour and sugar were removed and replaced with apple extract. P: cookies in which 5% (P05) and 10% (P10) of flour and sugar were removed and replaced with pomegranate peel extract.

		Without Substitution		With AE		With PPE	
	CON	W05	W10	A05	A10	P05	P10
All-purpose flour (g)	120	114	108	114	108	114	108
White Sugar (g)	60	57	54	57	54	57	54
Extract (g)	0	0	0	9	18	9	18
Baking Powder (g)	2	2	2	2	2	2	2
Margarine (g)	48	48	48	48	48	48	48
Water (g)	30	30	30	30	30	30	30

## Data Availability

Data are contained within the article and Supplementary Materials.

## Supplementary Materials

The following supporting information can be downloaded at https://www.mdpi.com/article/10.3390/molecules29051102/s1, Table S1: Water activity, moisture content, and hardness of cookies immediately after baking (t0) and after one month of storage (t1); Table S2: Total phenolic content (TPC) and DPPH as IC50 of cookies immediately after baking (t0) and after one month of storage (t1); Table S3: The 23 sensory descriptors used for the cookie evaluation.
