# Supplementary material for: Can a Fraction of Flour and Sugar Be Replaced with Fruit By-Product Extracts in a Gluten-Free and Vegan Cookie Recipe?
Source: Molecules. 2024 Feb 29;29(5):1102. doi: 10.3390/molecules29051102 (PMC10934159; doi:10.3390/molecules29051102)
Supplement: Supplementary file 1 [file molecules-29-01102-s001.zip › molecules-2855252-supplementary.pdf]

molecules

MDPI

Supplementary File of the manuscript

Table S1. Water activity, moisture content, and hardness of cookies immediately after baking (t0) and after one month of storage (t1).

		a_w		Moisture content (%)		Hardness (N)	
		**t0**	**t1**	**t0**	**t1**	**t0**	**t1**
	**CON**	0.25 ± 0.08	0.27 ± 0.09	4.5 ± 0.9	3.3 ± 1.1	23.4 ± 4.0	25.1 ± 2.7
	**W05**	0.25 ± 0.07	0.34 ± 0.03	3.8 ± 1.5	4.2 ± 0.4	23.0 ± 4.2	22.1 ± 3.6
	**W10**	0.29 ± 0.11	0.32 ± 0.06	4.8 ± 2.2	3.9 ± 0.5	25.5 ± 6.2	20.2 ± 5.8
	**A05**	0.24 ± 0.01	0.28 ± 0.03	4.7 ± 0.8	3.6 ± 0.5	29.4 ± 1.9	26.4 ± 4.1
	**A10**	0.16 ± 0.06	0.25 ± 0.05	2.8 ± 1.7	2.9 ± 0.4	33.5 ± 5.7	28.4 ± 4.0
	**P05**	0.22 ± 0.08	0.27 ± 0.05	3.8 ± 1.6	3.2 ± 0.7	41.0 ± 10.7	27.6 ± 6.4
	**P10**	0.24 ± 0.04	0.26 ± 0.02	4.1 ± 1.3	3.1 ± 0.3	35.6 ± 7.0	27.3 ± 6.8
p-value	*Sample*	n.s.		n.s.		*	
	Time	n.s.		n.s.		n.s.	
	*Sample*Time*	n.s.		n.s.		n.s.	

Results are expressed as means (n = 3) ± standard deviation.

*, ** and *** indicate significant differences by two-way ANOVA at $p < 0.05$, $p < 0.01$ and $p < 0.001$, respectively, for the samples. ns = not significant.

Table S2. Total Phenolic Content (TPC) and DPPH as IC_{50} of cookies immediately after baking (t0) and after one month of storage (t1).

		TPC (mgGAE/g)		DPPH (IC50 μg/mL)	
		**t0**	**t1**	**t0**	**t1**
	**CON**	0.75 ± 0.35	0.64 ± 0.35	25.6 ± 4.3	21.3 ± 5.9
	**W05**	0.61 ± 0.02	0.46 ± 0.14	50.4 ± 6.5	52.6 ± 2.6
	**W10**	0.59 ± 0.10	0.47 ± 0.12	46.9 ± 2.6	46.2 ± 8.8
	**A05**	0.92 ± 0.18	0.83 ± 0.07	28.9 ± 3.9	29.4 ± 1.6
	**A10**	1.30 ± 0.12	1.51 ± 0.15	20.7 ± 0.9	20.9 ± 2.0
	**P05**	6.15 ± 0.37	6.87 ± 0.17	3.1 ± 1.9	2.2 ± 0.3
	**P10**	9.77 ± 0.95	10.06 ± 0.69	1.2 ± 0.3	1.1 ± 0.1
p-value	*Sample*	***		***	
	Time	n.s.		n.s.	
	*Sample*Time*	n.s.		n.s.	

Results are expressed as means (n = 3) ± standard deviation.
*, ** and *** indicate significant differences by two-way ANOVA at $p < 0.05$, $p < 0.01$ and $p < 0.001$, respectively, for the samples. ns = not significant.

Table S3: The 23 sensory descriptors used for the cookies evaluation.

**Appearance (external surface)**	Homogeneity of colour	1 = Extremely irregular	9 = Extremely homogeneous
	Homogeneity of shape (round)	1 = Extremely irregular	9 = Extremely homogeneous
	Roughness of surface	1 = Extremely smooth	9 = Extremely rough
	Porosity	1 = Extremely compact	9 = Extremely porous
**Aroma**	Toasted-Burned	1 = Extremely slight	9 = Extremely intense
	Caramel	1 = Extremely slight	9 = Extremely intense
	Fruity	1 = Extremely slight	9 = Extremely intense
	Vanilla	1 = Extremely slight	9 = Extremely intense
	Butter	1 = Extremely slight	9 = Extremely intense
	Whole	1 = Extremely slight	9 = Extremely intense
**Texture/Mouthfeel**	Hardness	1 = Extremely soft	9 = Extremely hard
	Granularity	1 = Not at all granulose	9 = Extremely granulose
	Friability	1 = Not at all friable	9 = Extremely friable
	Flouriness	1 = Not at all powdery	9 = Extremely powdery
	Adhesiveness	1 = Not at all adhesive	9 = Extremely adhesive
	Astringency	1 = Not at all astringent	9 = Extremely astringent
**Taste**	Sweet	1 = Extremely slight	9 = Extremely intense
	Bitter	1 = Extremely slight	9 = Extremely intense
	Sour	1 = Extremely slight	9 = Extremely intense
**Flavour/Aftertaste**	Fruity	1 = Extremely slight	9 = Extremely intense
	Caramel	1 = Extremely slight	9 = Extremely intense
	Tosted	1 = Extremely slight	9 = Extremely intense
	Persistance	1 = Extremely slight	9 = Extremely intense
